# Dramatic response to infliximab in refractory neurosarcoidosis

**DOI:** 10.4103/0972-2327.70874

**Published:** 2010

**Authors:** Sreekanth Chintamaneni, Aarat M. Patel, Samuel B. Pegram, Hirenkumar Patel, Heidi Roppelt

**Affiliations:** Department of Rheumatology, Stony Brook University Medical Center, Stony Brook, NY, USA; 1 Rheumatic Disease Clinic of Houston, Houston, TX, USA

**Keywords:** Infliximab, neurosarcoidosis, tumor necrosis factor-α

## Abstract

Sarcoidosis is a systemic disease characterized by noncaseating granulomas in the involved organs. Neurologic manifestations involving the central and/or peripheral nervous system occur in about 5% of patients. Neurosarcoidosis is often refractory to conventional treatment and therefore more effective treatment options are needed. While the etiology of the disease is still unknown, there is now a better understanding of its pathogenesis on a molecular level. It is clear that tumor necrosis factor-α (TNFα) plays a pivotal role in the development of the granulomas and it is believed to be a key cytokine involved in the pathogenesis of the disease. Taking advantage of this better understanding of disease pathogenesis, anti-TNFα agents are being increasingly used to treat refractory sarcoidosis. We report a patient with refractory neurosarcoidosis who showed dramatic improvement in the clinical and radiological manifestations following treatment with infliximab; he suffered a relapse upon discontinuation of the medication.

## Introduction

Sarcoidosis is a systemic disease of unknown etiology that is characterized by noncaseating granulomas in involved organs. At initial presentation there can be involvement of a number of organs, but patients commonly present with hilar adenopathy, lung, eye, and/or skin involvement.[1] The diagnosis is suggested by the clinicoradiological findings and is secured by histological confirmation of the presence of noncaseating granulomas. Exclusion of other conditions which can produce similar pathology, including other autoimmune diseases, inhalation diseases, neoplasia, and a multitude of infections (particularly those related to mycobacteria and fungi), is imperative.[1]

Neurologic manifestations occur in about 5% of patients and both the central and peripheral nervous system can be affected. When this disorder is suspected, a search for extraneural sarcoidosis is warranted due to the difficulty of obtaining nerve tissue for diagnostic evaluation. There is no pathognomonic diagnostic test for neurosarcoidosis. Magnetic resonance imaging (MRI) can assist in excluding other diseases and be helpful in suggesting a presumptive diagnosis of neurosarcoidosis.[2]

No agents have been approved for the treatment of neurosarcoidosis. High-dose corticosteroids are often used for management despite the absence of controlled treatment trials suggesting their benefit. The adverse effects of steroid therapy, refractory disease, and primary contraindication to corticosteroids has led to the use of alternative therapies such as methotrexate, cyclophosphamide, cyclosporin, chlorambucil, chloroquine, radiotherapy, and hydroxychloroquine.[2] We report a case of a patient refractory to conventional treatment who was successfully treated with infliximab, with dramatic clinical and radiological improvement.

## Case Report

A 50-year-old white male developed numbness of the tongue, lips, and face. An MRI scan of the brain revealed an area of altered signal intensity in the left middle cerebellar peduncle. Further evaluation, including magnetic resonance angiography (MRA), transesophageal echocardiogram, and lumbar puncture, was normal. Serologic studies to assess for infectious etiologies and antinuclear antibody (ANA) were negative; the erythrocyte sedimentation rate was normal. The patient was presumed to have had a small cerebrovascular accident. His symptoms resolved spontaneously over 4 months.

Two years later, the patient again developed facial numbness with slurred speech, diplopia, and gait instability. MRI scan of the brain revealed an 8-mm enhancing lesion in the left cerebellum. Evaluation for an underlying malignancy with computed tomographic (CT) scan of the chest/abdomen/pelvis revealed no abnormalities and human immunodeficiency virus (HIV) testing was negative. Cerebrospinal fluid (CSF) analysis revealed elevated protein of 103 mg/dl (normal: 15–45 mg/dl) but was otherwise normal. CSF angiotensin-converting enzyme (ACE) level was not performed. A needle biopsy of the lesion revealed nondiagnostic atypical lymphoid tissue, and cultures for mycobacteria and fungi were negative.

A follow-up MRI done 3 months later revealed multiple new enhancing lesions in the cerebellar hemispheres. Extensive evaluation by a neurologist did not identify a cause. Serial MRI scans revealed spontaneous improvement in the size of the multiple enhancing lesions involving the cerebellar hemispheres, with corresponding clinical improvement. However, the patient had recurrence of gait instability and diplopia and a repeat MRI revealed progression of the cerebellar lesions, along with a dominant new lesion adjacent to the right lateral recess of the fourth ventricle.

The patient was referred to a tertiary medical center for further evaluation. He underwent a second needle biopsy which again was nondiagnostic, demonstrating only gliotic reactive tissue. Multiple studies were performed to exclude lymphoma and infectious etiologies. Evaluation by an ophthalmologist revealed no evidence of uveitis and the patient had no systemic symptoms or chest X-ray findings suggestive of sarcoidosis. The patient refused an open brain biopsy.

Based on the patient’s clinical presentation and MRI findings, and due to exclusion of other etiologies, the patient was considered to have a variant of neurosarcoidosis and was started on oral dexamethasone 4 mg daily. Both diplopia and gait improved on corticosteroids but the symptoms recurred later that year. The patient was then referred for a second opinion to another tertiary medical center, where they concurred with the tentative diagnosis of neurosarcoidosis. Dexamethasone was discontinued in favor of oral prednisone 25 mg daily in addition to pentoxifylline, with mild clinical improvement. Due to persistent symptoms, the patient’s prednisone was increased to 60 mg daily; this was followed by significant improvement in his symptoms. He was eventually tapered off prednisone and maintained on pentoxifylline, and follow-up MRI scans showed no interval change.

One year later the patient had a recurrence of the neurological symptoms, with slurred speech, ataxia, a tendency to fall, poor coordination of upper limb movement, and numbness on the left side of his body. MRI of the brain revealed numerous areas of abnormal enhancement in both cerebellar hemispheres, the brain stem, and the upper cervical cord; there were more confluent areas of enhancement within the left parietal lobe white matter tract and in the deep white matter tract of the occipital lobe. Two new 1.5-cm sized areas of enhancement in the pons and parietal lobe were also noted. Prednisone 60 mg daily was again initiated, with some improvement in his symptoms. However, his clinical picture did not return to baseline. The patient was then referred to a rheumatologist for treatment of refractory neurosarcoidosis.

**Figure 1:**
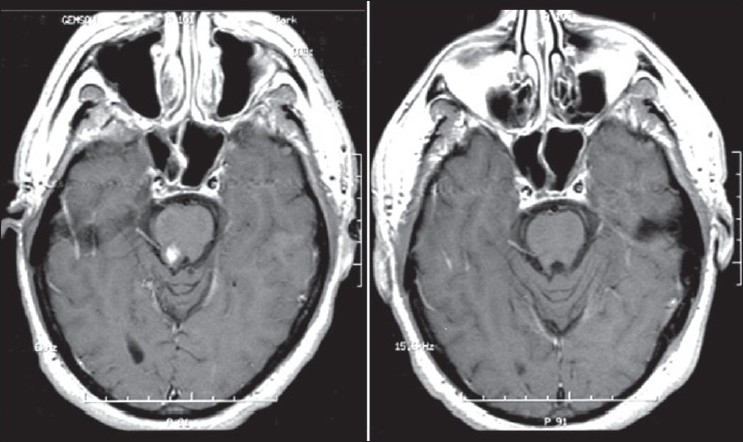
Axial T1-weighted postcontrast images demonstrating enhancing lesion in mid-pons 11/04 (left) and resolution of the lesion post infl iximab 9/05 (right)

**Figure 2:**
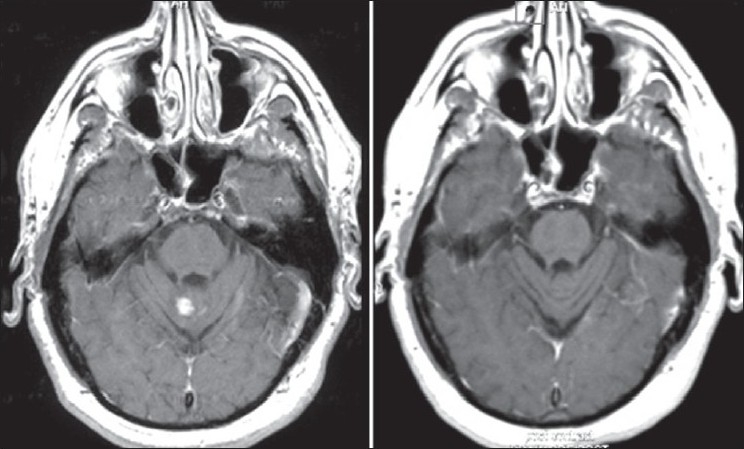
Axial T1-weighted postcontrast images demonstrating enhancing lesion in the vermis 8/06 (left) with resolution of the lesion post infl iximab 3/07 (right)

Given the burden of disease as shown on the MRI and the disabling nature of his symptoms, infliximab was started in January 2005. The patient had significant improvement in his gait, numbness, and slurred speech. After five infusions of infliximab, a repeat MRI revealed dramatic improvement, with resolution of lesions in multiple areas [Figure 1]. In August 2005, infliximab was discontinued as his medical insurance coverage had lapsed and the patient was started on hydroxychloroquine and methotrexate. He did fairly well for a year but then again developed neurological symptoms, with an MRI scan revealing new enhancing lesions in the left basal ganglia and vermis [Figure 2]. Infliximab was restarted and once again the patient had significant clinical improvement. An MRI scan several months later again showed resolution of the aforementioned lesions. The patient continues to do well on infliximab.

## Discussion

This case highlights the difficulty in diagnosing neurosarcoidosis, especially in patients in whom the disease is restricted to the central nervous system. Sarcoidosis is most easily diagnosed when there is ocular, cutaneous, or pulmonary involvement. Evaluation of this patient by an ophthalmologist failed to demonstrate evidence of uveitis or lacrimal gland involvement. Examination also failed to reveal any skin lesions consistent with sarcoidosis; pulmonary involvement was excluded based on CT imaging of the chest. Because histologic confirmation was not possible with biopsy from an extraneural site, the patient ultimately underwent two brain biopsies, both of which were nondiagnostic. Although this was likely due to sampling error, the biopsies did help exclude infectious etiology and malignancy, which were both considered at initial presentation.

In addition to infection and malignancy, there are other possible causes for this patient’s presentation and an exhaustive evaluation was undertaken to exclude other diseases. Multiple sclerosis, a demyelinating disease often characterized by optic neuritis and clinical relapses and remissions, was initially considered but then dismissed by all four neurologists who examined the patient, their opinions being based on the findings of additional imaging studies and his overall clinical course. The possibility of an autoimmune inflammatory disease was explored but autoantibodies, including ANA and anti-neutrophil cytoplasmic antibodies (ANCAs), were uniformly negative. Furthermore, examination by a rheumatologist revealed little clinical evidence to support a diagnosis of systemic lupus erythematosus, Wegener’s granulomatosis, or Behcet’s disease. In addition, MRA of the head and neck did not support a diagnosis of vasculitis which was another possibility. Only after careful exclusion of the aforementioned conditions by multiple specialists, was neurosarcoidosis diagnosed in this patient.

The etiology of sarcoidosis is unknown. Current evidence suggests that the disease is a result of exposure to an undefined yet persistent antigen, with a subsequent cell-mediated hypersensitivity response in persons with a genetic or inherited predisposition.[1] It has also been suggested that sarcoidosis may not result from a specific exposure but rather from an abnormal host immunologic response to one of several exposures.[3]

Among the cytokines implicated in the pathogenesis of sarcoidosis are interferon-γ (IFN-γ), interleukin-2 (IL-2), and tumor necrosis factor-α (TNFα). The role of TNFα in the pathogenesis of sarcoidosis has been studied extensively. TNFα plays a pivotal role in the development of granulomas and is thought to play a key role in mediating the inflammation among the numerous cytokines involved in the pathogenesis of sarcoidosis.[4,5] It has been found to be released by activated alveolar macrophages of patients with sarcoidosis and this release is significantly higher in patients with active sarcoidosis than in those with inactive disease.[6,7] Furthermore, patients refractory to treatment with corticosteroids tend to have high levels of TNFα in bronchoalveolar lavage fluid.[8] Based on this data, TNFα-blocking therapy has been used to treat refractory sarcoidosis but has given variable results.[9–11]

Three anti-TNFα agents are currently approved for treatment of rheumatic diseases: etanercept, a soluble receptor fusion protein; adalimumab, a fully human monoclonal antibody; and infliximab, a humanized mouse monoclonal antibody. TNFα inhibition with infliximab, adalimumab, and etanercept have all been proven to be effective in the treatment of rheumatoid arthritis, but their efficacy in granulomatous diseases, including sarcoidosis, is variable. Experience with adalimumab in sarcoidosis is limited to a few case reports, although a clinical trial is currently recruiting patients to study its efficacy in sarcoidosis of the skin.[12] Etanercept has been studied prospectively in refractory ocular sarcoidosis, with disappointing results.[9] Also, a phase-II treatment trial of etanercept for stage II or III progressive pulmonary sarcoidosis was terminated early due to excessive treatment failures in 11 of 16 patients experiencing worsening of their disease.[13]

The anti-TNFα agent infliximab has been used for the treatment of refractory sarcoidosis and has proven to be effective in many of its disease manifestations: lupus pernio, uveitis, hepatic sarcoidosis, cutaneous disease, and myositis.[10] Infliximab has been studied prospectively in pulmonary sarcoidosis, with modest improvement in forced vital capacity (FVC) compared to placebo.[14] Its efficacy in the treatment of extrapulmonary sarcoidosis was assessed as a secondary endpoint in the same study and infliximab was found to be beneficial compared to placebo in patients already receiving corticosteroids.[15] In addition, several case reports have shown remarkable efficacy in refractory neurosarcoidosis.[11,16–18] Based on a limited number of small studies, case series, and case reports, it is speculated that infliximab has greater efficacy than etanercept in the treatment of sarcoidosis, although this requires further investigation.[19]

The patient described in this report was treated with high-dose corticosteroids, methotrexate, pentoxifylline, and hydroxychloroquine but showed only minimal improvement. Infliximab resulted in dramatic clinical and radiologic improvement as described by his MRI findings. Furthermore, withdrawal of the medication for nonclinical reasons and reintroduction of therapy resulted in worsening and improvement in his condition, respectively. Because TNFα is believed to play a significant role in the pathophysiology of sarcoidosis, anti-TNFα agents are a mechanistically logical choice for therapy. This patient’s significant response to the anti-TNFα agent infliximab shows the need for further investigation into its use in neurosarcoidosis.
